# Evolution of Properties of High-Strength and High-Mg-Content CuMg Alloys After Being Subjected to Single Operation 50% Deformation in Hot and Cold Upsetting Tests

**DOI:** 10.3390/ma17225467

**Published:** 2024-11-08

**Authors:** Paweł Strzępek, Małgorzata Zasadzińska, Piotr Noga, Tomasz Skrzekut

**Affiliations:** Faculty of Non-Ferrous Metals, AGH University of Krakow, al. Mickiewicza 30, 30-059 Krakow, Poland; malgozas@agh.edu.pl (M.Z.); pionoga@agh.edu.pl (P.N.); skrzekut@agh.edu.pl (T.S.)

**Keywords:** CuMg, copper alloys, hot deformation, cold deformation, upsetting test, non-ferrous metals

## Abstract

Since most hot and cold metal-forming processes originate from various casting processes, it is important to test their susceptibility to the deformation of new materials. Cast rods of CuMg alloys with a Mg content of 2, 2.4, 2.8, 3, 3.2, 3.6, and 4 wt.% were obtained in the continuous casting process with pure copper as a reference material in order to obtain information on the material’s ability to withstand 50% deformation. The materials in the as-cast state were subjected to solutioning, cold drawing, and recrystallization. After each process, samples were taken and subjected to upsetting tests with 50% deformation applied in a single operation. Additionally, materials in the as-cast state were subjected to upsetting tests at 700 °C. The hardness and electrical conductivity of each sample were analyzed. Selected samples were subjected to microstructural analysis. The obtained results show an increase in hardness from 46 HB to 90–126 HB, and a further increase to 150–190 HB with a quasi-linear decrease of electrical conductivity, which proved the influence of solid-solution and strain hardening, respectively. The microstructural analysis proved that such deformation does not cause microcracks. Furthermore, in the case of CuMg up to 3 wt.% of Mg, the alloying additive completely dissolved after solutioning.

## 1. Introduction

Conventional metal-forming processes are based on continuous or semi-continuous casting processes followed by hot forming (rolling, extrusion, or forging usually preceded by extrusion) or cold forming (rolling, drawing, or stamping) with either machining or plating to obtain the final product [1,2,3]. There are alternatives such as near net shaping manufacturing, which proposes the continuous casting of semi-final products that are usually small, similar to the final product dimensions, and the subsequent hot forging with finishing [4]. This approach eliminates the necessity of extrusion before the final metal-forming process.

The applications of non-ferrous metals, being the products of various metal-forming processes, are limitless in the present world and include, among others, the aviation and space industry, the automotive industry, military applications, medical and consumer goods, heat exchangers, electronics and electrical conductivity, and many others [5,6,7]. Many of these require the materials to be of both high-strength and high electrical conductivity. Therefore, the use of steel for high-strength applications is often limited by its extremely low electrical conductivity, and thus, steel alloys are mostly used as construction materials or cores to increase the strength of overhead lines. It would be impossible to use pure copper as an overhead cable due to its weight-to-strength ratio, and on the other hand, it would be impossible to use just steel as a conductor in such lines due to their electrical conductivity-to-weight ratio [8,9,10]. Similarly, it would not be possible to use steel for trolley wires, and it would be complicated to use aluminum or aluminum alloys due to their low abrasive resistance, which would result in the constant need for wire replacement. Some of the ways of increasing the mechanical or functional properties of materials are non-conventional metal-forming processes such as the extrusion process with rotating die (KOBO) [11], accumulative angular drawing (AAD) [12], close die precision forging [13], die-less wire drawing [14], ultrasonic assisted wire drawing [15], or manufacturing metal matrix composites (MMC) [16]. On the other hand, pure metals such as copper or aluminum are sufficient among many applications where the electrical or thermal conductivity is of higher importance than the strength of the material [8,17,18]. However, in order to find a balance between the mechanical properties and the electrical or thermal conductivity, it is necessary to implement non-ferrous alloys. According to the Nordheim’s rule of mixtures, introducing foreign particles of different atom diameter during alloying would decrease the electrical conductivity of the matrix material even if the alloying element itself has higher electrical conductivity [19].

The most common, best studied, and oldest copper alloys are brass, where the main alloying element is zinc [20], and bronze, where the main alloy additive is tin [21]. There are, of course, grades of brass and bronze alloys that, apart from their main alloying elements, consist of a substantial amount of other additives such as Pb, Al, Ni, etc.; their purpose is either to increase plasticity, workability, and machinability, or to increase their mechanical properties [18,21,22]. In recent years, many other copper-based alloys have been designed, studied, and manufactured, such as CuNiSi alloys [23], CuAg alloys [24], CuSc alloys [25], CuCrZr alloys [26], and many others [18]. According to the authors [23,24,25,26], the abovementioned alloys are all described as high-strength due to being copper-based, and with limited alloying additives; their electrical conductivity is also at a reasonable or high level. However, the common use of these alloys is limited due to either the high price of alloying additives or their low availability [27].

The demand for wrought high-strength copper alloys is increasing due to the large amount of new applications such as 3D printing nozzles, welding tips, high-pressure heat exchangers and radiators, or ITER divertors (International Thermonuclear Experimental Reactor). Such applications require materials with high electrical and thermal conductivity and high strength as they face extreme working conditions. Therefore, it is important to conduct research on the alloys with cheaper alloying additives, which would still provide high mechanical properties and electrical conductivity. An alternative might be CuMg alloys with a higher than commercial Mg content, which, at the moment is at the maximum of 0.7 wt.% [28]. Commercially used CuMg alloys were studied by Calvillo et al. [29], who provided data proving that the ultimate tensile strength (UTS) of these alloys with 0.2 and 0.5 wt.% of Mg can increase from 170 MPa and 210 MPa to 550 MPa and 740 MPa, respectively, after 16 passes using an equal channel angular press. Recently, studies have been published on CuMg alloys with a higher than commercial Mg content; i.e., even above 2 wt.%., Gorsse et al. [30] studied the possibility of the precipitation hardening of CuMg alloys and showed evidence that precipitation hardening is very effective in the case of two-phase CuMg alloys, since materials subjected to both strain hardening and ageing can reach a Yield Strength (YS) of over 1000 MPa. However, it was also proven that two-phase alloys consist of excessive amounts of α + β-phase above 3.6 wt.% of Mg, which causes brittleness and the existence of microcracks after subjecting the as-cast materials to cold deformation in the upsetting tests [31]. Such microcracks and brittleness might limit the possibility of cold metal-forming without prior heat treatment.

Therefore, when new materials such as high-Mg-content CuMg alloys are in question, it is important to assess their susceptibility to deformation in various states, which is the main purpose of this paper. The objective is to verify the deformability of CuMg alloys obtained in the as-cast state by subjecting them to solutioning (causing supersaturation), prior cold deformation, recrystallization, and both cold and hot deformation in the uniaxial upsetting tests. Upsetting was chosen as it is a mechanical test that evaluates the behavior of metals under compressive stress. It determines the workability of materials as metals that can withstand large amounts of deformation without cracking are considered more workable, which is especially important when selecting materials for processes like forging. The conducted tests will also provide information on the strain hardening of tested materials being the effect of cold deformation [32]. Therefore, this paper will provide information on the response of newly developed alloys to deformation regarding the evolution of its properties and the existence of defects.

## 2. Materials and Methods

### 2.1. Chemical Composition of Selected Materials

The materials selected to obtain the main goal of the paper were cast rods from a laboratory horizontal continuous casting line built by Termateal for AGH University of Krakow (Termetal, Piekary Śląskie, Poland). The materials were obtained with identical process parameters to maintain the stability and purity of the experiment. The batch materials for the casting processes were ETP-grade copper of a declared purity of minimum 99.9 wt.% and magnesium of a declared purity of 99.99 wt.%. The diameters of the cast rods were 14 mm and the casting speed was 0.1 m/min in each case. However, the continuous casting process is not the main focus of this paper and more detailed information on the construction of the furnace and process parameters are available in the authors’ previously published paper [3]. The chemical composition of materials was selected based on the phase diagram of CuMg alloys [33] to represent both single-phase α alloys and two-phase α + β alloys. Additionally, pure copper was also casted as reference material. The verification of chemical composition is especially important since the melting temperature of Cu is slightly lower (1084 °C) than the boiling point of Mg (1091 °C), and thus the evaporation of alloying additive could occur [31]. In order to overcome this problem, the amount of Mg in the case of all casted alloys was 5% higher than the desired amount. The nominal chemical compositions are presented in Table 1.

According to the CuMg phase diagram [33], up to approximately 3 wt.% of Mg, the alloys are single-phase and two-phase above 3 wt.% of Mg. Therefore, the study examines three alloys each with additional alloys casted at the 3 wt.% of Mg boundary and pure copper.

The chemical composition of the obtained materials was measured and analyzed with an arc spark spectrometer SPECTROTEST TXC35 (SPECTRO Analytical Instruments, Kleve, Germany). The device uses optical emission. The analyzed sample is vaporized from the surface with an electrode by an arc spark discharge. While vaporized, the atoms and ions are excited into the emission of radiation. The radiation is passed to the optics of the spectrometer through optical fiber, where it is dispersed into spectral components. Based on the wavelengths and the intensity of radiation the data are recalculated using a stored set of calibration curves and the obtained results are presented as percentage concentration. Each rod was machined to the axis of the rod’s cross-section and by conducting five measurements on each of the rods the average values of Cu and Mg were calculated. The elements other than Cu and Mg were omitted in the further analysis as their amount was insignificant.

### 2.2. Upsetting Tests

**Figure 1:**
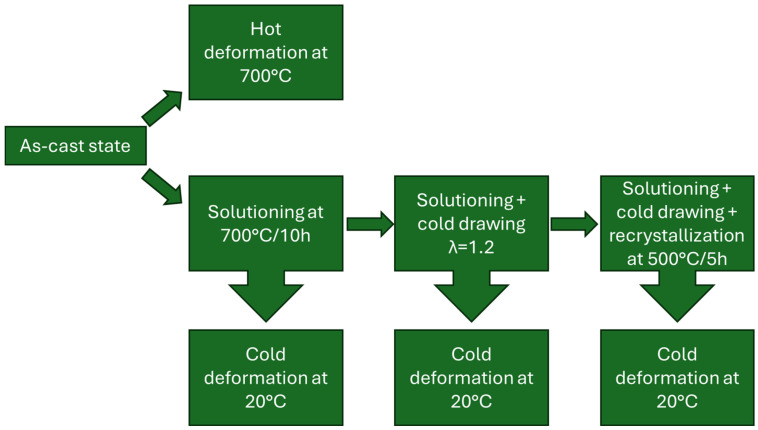
The schematic research program.

Upsetting is currently widely used in metal forming to improve the mechanical properties of metal products; however, there are several disadvantages regarding the process such as the formation of cracks [31,32]. The materials obtained in the as-cast state were subjected to several state modifications as presented in Figure 1 in order to obtain information on how to prevent formation of said cracks. Regarding the cold deformation of CuMg alloys in the as-cast state, an extended analysis was conducted in the previously published paper [31], and it was therefore decided to focus on new data instead of repeating the same experiments. Due to planned cold deformation in the drawing process and the resulting diameter reduction it was important that the initial casted rod had a diameter of 14 mm. It allowed for all the samples to have the same cylindrical shape and identical dimensions.

**Figure 2:**
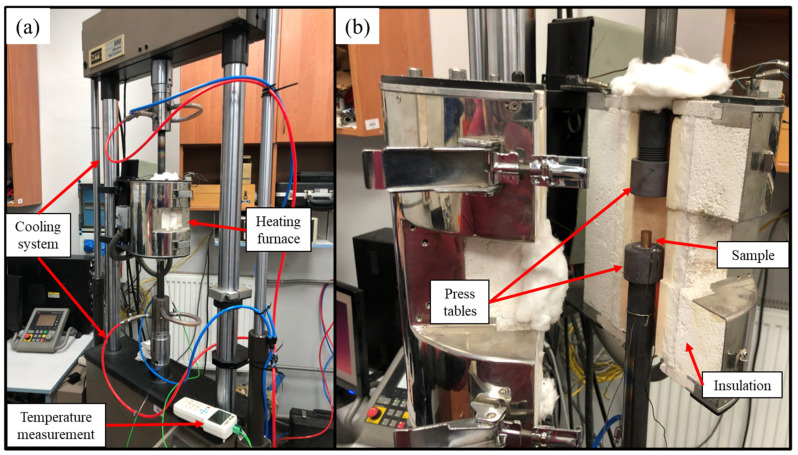
The overview of the hot upsetting test stand; (**a**) the view with a closed heating furnace; (**b**) the view with an open heating furnace and sample before upsetting placed on a press table.

The samples after each of the depicted state modifications are in Figure 1. Heat treatment processes were conducted using the resistance furnace (Czylok, Jastrzębie-Zdrój, Poland) and/or prior deformations were subjected to cold deformation in upsetting tests on the MTS 880 hydraulic press (MTS Systems Corporation, Eden Prairie, MN, USA). The samples in the as-cast state were additionally subjected to hot deformation at 700 °C by applying the heating furnace (Severn Science Ltd., Bristol, United Kingdom) during the upsetting test as presented in Figure 2. The extensions for press tables were made of quenched steel with the cooling medium applied inside to prevent the boiling of the hydraulic oil. The surface of the press tables during both hot and cold upsetting tests was covered with boron nitride as lubricant, which is known to withstand the temperatures of up to 1000 °C in the air atmosphere [34]. The temperature of the furnace chamber was constantly monitored with thermocouples. The samples during hot upsetting were heated along with the furnace (at the rate of approximately 5 °C/min) and when the temperature of 700 °C was obtained each sample was held at that temperature for 1 h prior to deformation to ensure that the whole sample was heated homogenously. The samples were cylindrical with a diameter of 10 mm and a height of 15 mm and the applied test velocity was 1 mm/min. The deformation applied regardless of the state of the material was 50% in a single operation. The 50% deformation was chosen for two reasons. Firstly, it provides sufficient amounts of data for further analysis using the Hollomon equation. Secondly, in [31] it was discussed that during cold deformation of the two-phase alloys in the as-cast state the cracks were visible when subjecting them to 50% of strain. The point was to determine whether it would still be the case after solutioning at 700 °C.

Due to this research program, there were eight different materials tested in the initial state, and after, the application of four various types of deformation (hot deformation and cold deformation of three various states of material). Overall, thirty-two different types of samples were subjected to deformation, the properties analyses and their evolution as well as the microstructural analyses are described in the following subsections.

### 2.3. Microstrutural Analysis

The microstructural analysis was conducted on selected samples that would represent pure copper, single-phase alloy (CuMg2), two-phase alloy (CuMg4), and the alloy that is approximately at the boundary between phases (CuMg3) in order to reduce the amount of data. The analyses were conducted using Olympus GX51 inverted metallurgical optical microscope (OM) (Olympus, Tokyo, Japan) and Hitachi S-3400N scanning electron microscope (SEM) (Hitachi Ltd., Tokyo, Japan). Regarding the CuMg alloys, the refining of grain size according to the Hall–Petch equation and the presence of fine and evenly distributed Mg-rich precipitates might significantly increase mechanical properties. However, an excessive amount of precipitates and too fine grain might cause brittleness, which could result in lower plasticity and lower resistance to dynamic impact toughness. By using OM, it was possible to determine the grain size at different states of materials after deformation and the prospective existence of macroscopic cracks. Samples were polished using Struers Labopol polishing unit (Struers, Ballerup, Denmark) and the etching solution was FeCl3-HCl (5 g of Iron(III) chloride, 10 cm^3^ of Hydrochloric acid, 100 cm^3^ of ethanol). By using SEM, it was possible to determine the presence of microcracks at the α + β eutectic, which would be impossible to see without such high magnification. For the SEM analysis, samples were flat, polished, and perpendicular to the electron beam. The accelerating voltage was set at 10 kV, the working distance was set to 10 mm, and the magnification was 3000×.

**Figure 3:**
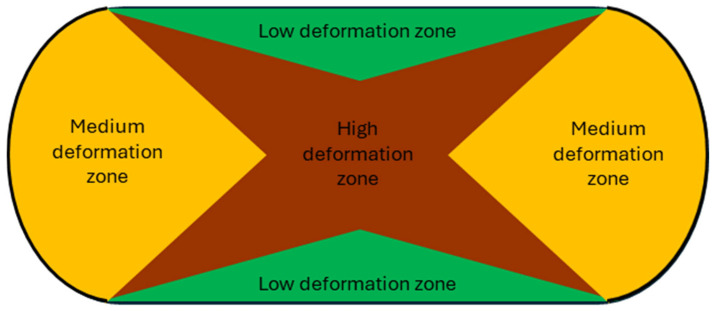
A schematic cross-section of deformation inhomogeneity after the upsetting test; own work is based on data from [32,35,36].

It is generally accepted that strain in an upsetting test is not perfectly homogeneous. The analyses of microstructure and further discussed macroscopic properties (hardness and electrical conductivity) were done at a high deformation zone, close to the middle of each sample as presented in Figure 3 [32,35,36].

### 2.4. Hardness and Electrical Conductivity

Upsetting is described as one of the forging methods [37], which, especially in higher temperatures, may eliminate internal pores of the cast [38,39]. However, applying a high temperature may also affect the electrical conductivity of alloys [18]. On the other hand, upsetting at the ambient temperature, regardless of the state of materials, would provide cold deformation, and thus strain hardening, which would affect the hardness of tested materials [31]. In order to assess the evolution of properties after each sample was subjected to deformation, both hardness and electrical conductivity were measured.

The electrical conductivity of materials was measured with a non-destructive test (NDT) using an eddy current measuring device SigmaTest 2.069 (Foerster Instruments Inc., Pittsburgh, PA, USA). Prior to each measurement, samples were placed in the air-conditioned laboratory to maintain constant temperature and stabilize the thermal state of the samples. This is important as the electrical conductivity of metals and alloys decreases with the increase of temperature [40]. It is especially important when assessing samples subjected to the various heat treatment processes. The frequency was set at 60 KHz and each sample was subjected to five measurements.

Hardness was determined with the Brinell method, which provides better results regarding high-strength hardened alloys. The results might have high standard deviation if a microhardness assessment method such as the Vickers method was used. The measurements were conducted with a Nexus3001 hardness tester (Innovatest Europe BV, Maastricht, The Netherlands), that has a test force accuracy of 0.5% and a display resolution of 0.1 HB. The applied load was 187.5 kgf (approximately 1840 N) with 10 s of indenting time and each sample was subjected to five measurements.

## 3. Results and Discussion

### 3.1. Chemical Composition of Selected Materials

The chemical composition of samples was selected based on a CuMg phase diagram [33] to represent single-phase α alloys (CuMg2, CuMg2.4, CuMg2.8), two-phase α + β alloys (CuMg3.2, CuMg3.6, CuMg4), and an alloy approximately at the boundary between these two groups (CuMg3) with a reference material of pure copper. The results of chemical composition analyses are presented in Table 2. The arc spark spectrometer used for the analysis provides results of much more elements; however, since there were no alloying additives other than Cu and Mg, their measured content was 0 ppm or close to 0 ppm. Therefore, it was decided to present their sum as balance since it was not meaningful. The average results presented in Table 2 include the dispersion of the measurements. The measured values were both repeatable and consistent with the nominal chemical composition presented in Table 1 in the previous section. Due to the evaporation of magnesium [31], even though the amount of alloying additive was 5% higher than nominal as mentioned in the previous section, the measured values were slightly lower than desired but in the desired nominal range.

Furthermore, due to low values of dispersion of Mg in Table 2 it is assumed that the materials did not exhibit macrosegregation, which could influence the further research regarding upsetting tests and the evolution of properties [41,42,43]. Bearing that in mind, the samples were subjected to further analyses.

### 3.2. Upsetting Tests

**Figure 4:**
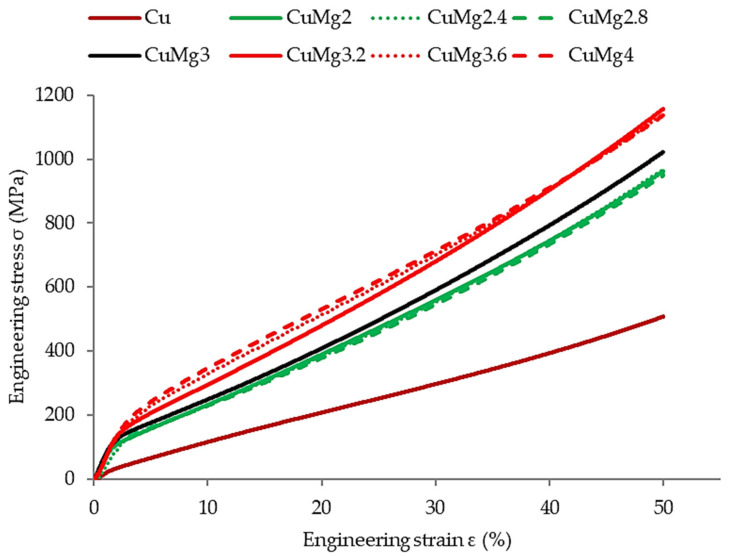
The stress–strain relation of supersaturated samples subjected to the cold upsetting test.

**Figure 5:**
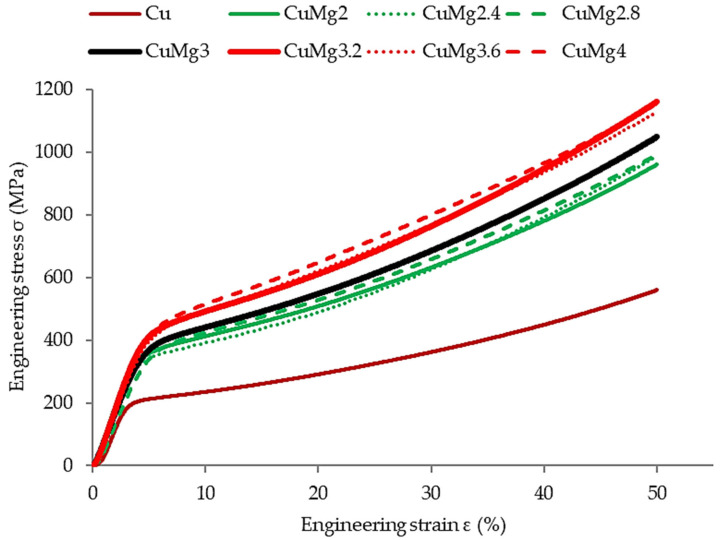
The stress–strain relation of supersaturated and cold drawn samples subjected to the cold upsetting test.

**Figure 6:**
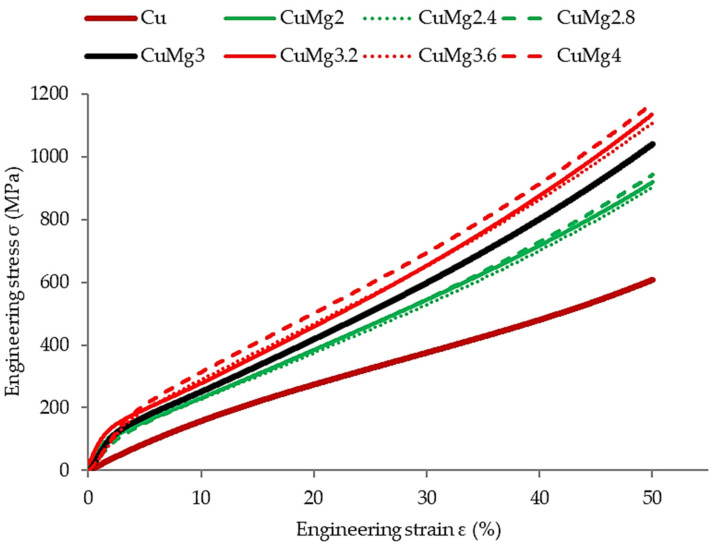
The stress–strain relation of supersaturated, cold drawn, and recrystallized samples subjected to the cold upsetting test.

**Figure 7:**
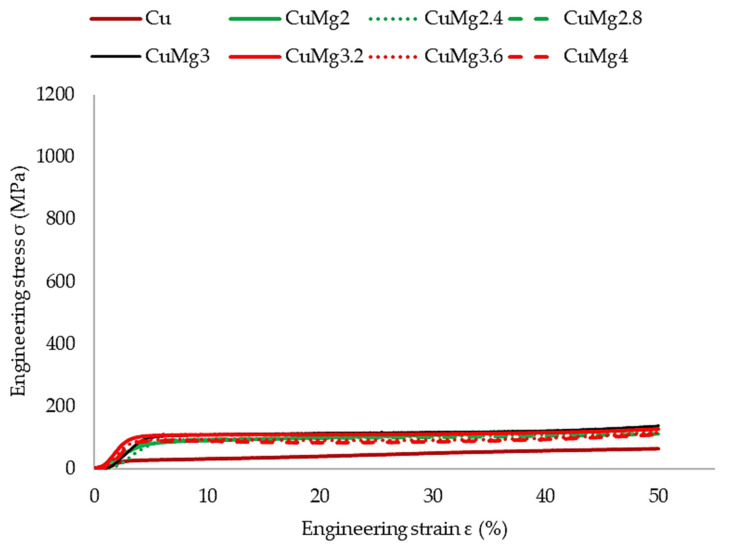
The stress–strain relation of as-cast samples subjected to the hot upsetting test.

In order to verify the response of the materials to cold deformation in various states, the samples were subjected to 50% upsetting tests. The results of these tests in the form of stress–strain curves are presented in Figure 4 (supersaturated samples), Figure 5 (supersaturated and cold drawn samples), and Figure 6 (supersaturated, cold drawn, and recrystallized samples). After cold upsetting was performed, the press tables extensions with cooling systems and a heating furnace were attached to the press and hot deformation was applied to the samples at 700 °C. The results of hot upsetting are presented in Figure 7.

In order to ease the analysis of the presented results in all of the cases (Figure 4, Figure 5, Figure 6 and Figure 7), pure copper is marked with a brown color, the single-phase CuMg alloys are marked with a green color, the two-phase CuMg alloys are marked with a red color, and the CuMg3 alloy, which is in between according to the CuMg phase diagram [33], is marked with a black color. A strain of 50% was applied as it provides a sufficient amount of data for further analysis [31]. Cold deformed samples exhibited much higher stress recorded, regardless of the state of material than samples subjected to hot deformation. This is in agreement with other papers published on upsetting tests [44,45,46] where the authors claim that lower temperatures allow higher plastic strain energy to be imparted even at a small amount of strain. The effect of solid-solution hardening is undeniable as all alloys required higher stress to be applied in order to obtain 50% deformation than pure copper [18]. Regarding CuMg alloys in comparison with pure copper, which is tested in the current paper, the increase is approximately twofold. Even during hot deformation, the stress required to obtain 50% of strain was two times higher in the case of all CuMg alloys than for pure copper.

The collective data are presented in Table 3. Since the stress–strain curves are almost perfectly represented in four clusters (Cu, single-phase alloys, CuMg3, two-phase alloys), the data are divided into these four groups.

The existence of said clusters must be directly connected to the amount of α + β phase in the alloys that should dissolve in elevated temperatures and be prevented from occurring during cooling due to rapid quenching [47]. This is especially since when no heat treatment was applied in [31] and the alloys were subjected to upsetting in the as-cast state, this phenomenon did not occur. What needs to be noted is the fact that the applied heat treatment did not affect pure copper in the way the alloys were affected. The stress needed to obtain 50% of cold deformation of pure copper increased after recrystallization, whereas in the case of single-phase CuMg alloys it decreased significantly, and in the case of CuMg3 and two-phase alloys it remained at similar levels as after cold drawing.

According to the presented stress–strain curves, the elastic deformation region and YS increased significantly after cold drawing in regard to just supersaturated samples. The YS decreased again after applying recrystallization heat treatment, and the starting point of plastic deformation was at the level similar or slightly lower than the supersaturated samples. This suggests that the recrystallization parameters were selected properly, the grain size should decrease, and mechanical properties of materials should decrease as well [48,49].

**Figure 8:**
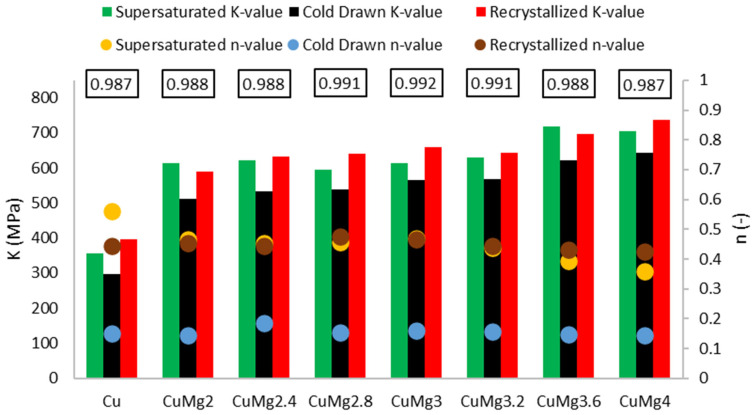
The collective calculated data according to the Hollomon equation; K—strength coefficient; n—strain hardening exponent; R^2^ values in rectangles—coefficient of determination.

For further analysis of the cold deformed samples, the strain hardening exponent and the strength coefficient proposed by Hollomon [50] were calculated according to Equation (1) and presented collectively in Figure 8,

(1) $$\sigma_{s}=K\times {\varepsilon_{t}}^{n}$$

where σ_s_ is the deformation resistance, K is the strength coefficient, ε_t_ is the true strain, and n is the strain hardening exponent.

For each of the tested materials, an average R^2^ coefficient of determination values is presented at the top of the graph in Figure 8. These calculated values show very high levels of accuracy of prediction as they are close to or above 0.99 in the range of 0 to 1.

The calculated n-values, which represent the formability of materials, show that after just solutioning the formability decreases from 0.56 for Cu to 0.36 for CuMg alloys with the highest Mg content. Therefore, it suggests that if it was technologically possible pure copper would reach even higher mechanical properties than CuMg alloys if given enough strain. These data are in agreement with results obtained for the CuMg alloys in the as-cast state [31]. The differences disappear after applying cold drawing, or cold drawing and recrystallization. The n exponent is drastically lower after cold drawing, in comparison to supersaturated or recrystallized samples, which shows the limitation of deformability of copper and copper alloys.

The K-value represents the theoretical true strength of the materials if a true strain equal to 1 would be applied. Since cold drawing applies initial strain and obtains higher YS value its maximum level is therefore limited. This is why the K-values after cold drawing are the lowest of all the tested samples. However, in comparison with Cu, the increase of K-values is between 72% and 116% depending on the Mg content, while for the supersaturated and recrystallized samples it is in the range of 71–97% and 48–86%, respectively. The changes in calculated values show that CuMg alloys are highly responsive to heat treatment and their properties can be altered by it. However, higher mechanical properties are limited by lower formability [31,51].

The authors in [31,52] observed that alloys with higher amount of α + β phase when subjected to cold deformation in the as-cast state tend to crack. The macroscopic result of these cracks was visible on the upsetting stress–strain curves as the stress at some point stopped rapidly increasing—there was a breaking point visible [31]. The authors in [53] claim that by applying the heat treatment the fracture strength in compressive tests can be increased by almost 80% and fracture strain by almost 100%. Since no sudden changes throughout the stress–strain curves occurred for any of the alloys studied in this paper it might suggest that no significant cracks occurred during the conducted tests and thus the applied heat treatment resulted in dissolving most or a significant amount of precipitates. This will be verified further in the next subsection.

### 3.3. Microstructural Analysis

**Figure 9:**
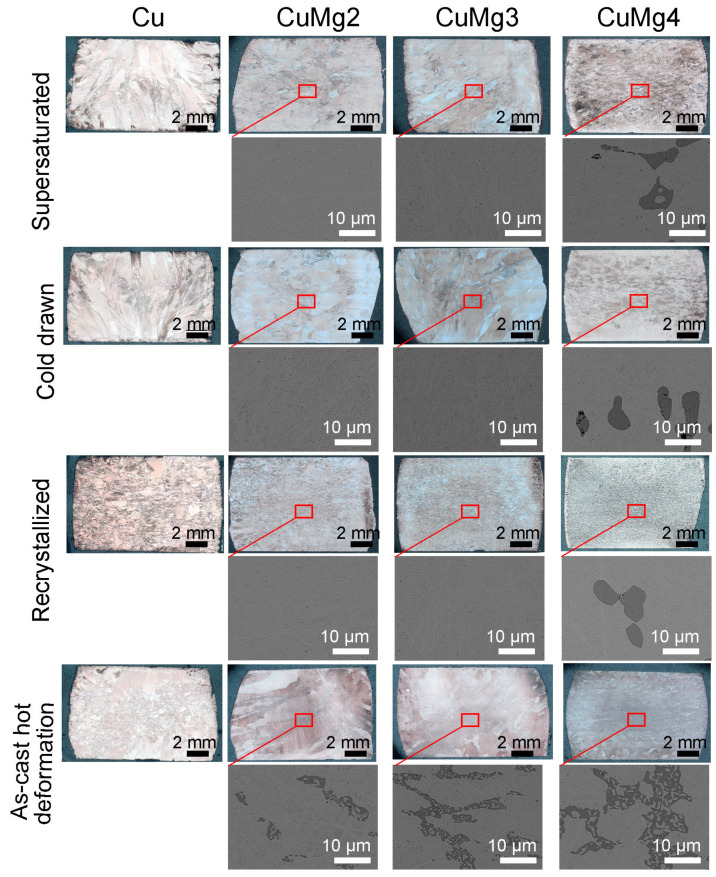
The microstructural analysis of the selected samples after the upsetting tests.

Figure 9 depicts the microstructure of the selected samples after the upsetting tests depending on the Mg content and the applied state modifications (the heat treatment and initial cold deformation). Additionally, the microstructure of pure copper is also presented for reference. Regardless of the magnesium content, no cracks were found in any of the samples after the heat treatment was applied prior to upsetting. This proves the high plasticity of all samples and the possibility of shaping these materials without loss of cohesion as opposed to samples subjected to cold deformation in the as-cast state [31]. Using optical microscopy, it can be observed that with increasing magnesium content, the grain size decreases. This phenomenon may be caused by the formation of more numerous crystallization nuclei in the form of intermetallic phases. This may make the grains smaller, as more crystals are formed at the early stages of solidification. Additionally, a larger number of these phases hinders the diffusion of atoms in the solid state, which inhibits further grain growth [54]. Such reasoning may be confirmed by assessing the SEM images of samples subjected to upsetting at 700 °C. In the case of SEM analysis of cold deformed samples, it can be stated that subjecting CuMg alloys with 2 and 3 wt.% of Mg to solutioning and thus in the supersaturated state caused the phases rich in Mg to be completely dissolved. Consequently, these phases are also dissolved in the case of cold drawn and recrystallized samples. Whereas, in the case of CuMg4 alloys, the dissolution was only partial and the dark α + β phase is clearly visible. According to the CuMg phase diagram, the second phase is Cu2Mg. The darker color is the result of the SEM analysis, which was conducted using backscatter electrons (BSE), and thus, the elements or phases with the lower atomic number are darker [52]. Additionally, the Mg-rich phases of CuMg4 samples were fragmentized after subjecting them to cold upsetting when the sample was in the cold drawn state. It was not present in the supersaturated or recrystallized state. It might suggest that two-phase CuMg alloys may have limited deformability, even after prior solutioning was applied. Out of all the analyzed states of materials the grain size was the smallest in the recrystallized state, regardless of the Mg content. The reason for that may be the fact that such heat treatment preceded by cold deformation leads to significant grain refining in the materials microstructure. It is caused by the formation of dislocations and recrystallization that leads to new, fine grains being formed [55]. The process is further enhanced by the addition of Mg, which inhibits the grain growth and stabilizes the fine-grained structure.

### 3.4. Hardness and Electrical Conductivity

**Figure 10:**
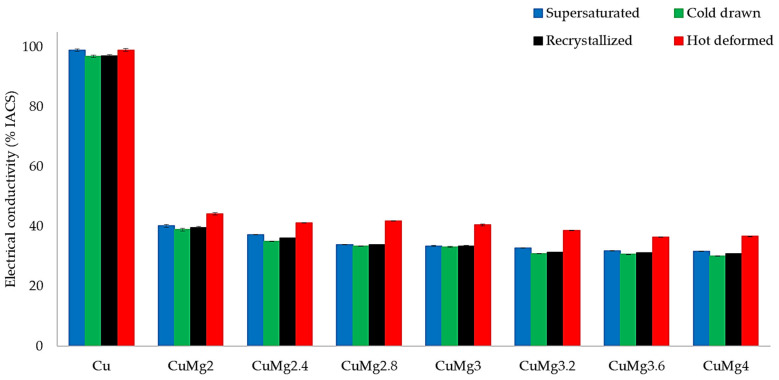
The collective average values of electrical conductivity expressed in % IACS.

The analysis of the macroscopic properties of CuMg alloys subjected to cold deformation in various states was focused on electrical conductivity expressed as % IACS (International Annealed Copper Standard) and hardness measured using Brinell’s method. IACS percentage is the percentile value where 100% is equal to the electrical conductivity of pure copper in the annealed state. Figure 10 presents the results of electrical conductivity measurements after subjecting the samples to upsetting tests. The standard deviation of conducted measurements is marked as error bars; however, in many cases it was so low that it is barely visible. The values for pure Cu are always around 100% IACS, while the values for CuMg alloys decrease quasi-linearly with the increase of Mg content according to Nordheim’s rule of mixtures [19]. The measured values for cold deformed samples are almost identical, while hot deformed are significantly higher. This was confirmed for many non-ferrous metals and alloys and is especially visible when cold deformation is higher than 40%, regardless of the orientation of measurement (axial/radial) [56]. It also explains why the cold drawn samples were recorded with the lowest electrical conductivity values.

The authors in [57] explain the difference in electrical resistivity between the deformed and recrystallized samples and the difference in stacking fault energy. They claim that the difference between the values decreases as the stacking fault energy increases. Since, in this study, the electrical conductivity of recrystallized samples is almost identical to cold drawn and supersaturated samples it would suggest that the stacking fault energy is significantly high.

**Figure 11:**
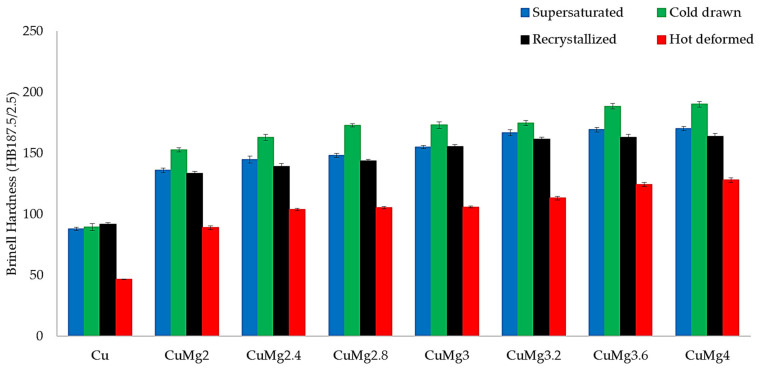
The collective average values of Brinell hardness.

The differences between various states were not that substantial in terms of electrical conductivity; however, in terms of hardness, the measured values varied significantly after subjecting the samples to cold upsetting at 50% strain. The calculated average values with standard deviation marked as error bars are presented in Figure 11. Regarding pure copper, all cold deformed samples were measured at approximately 90 HB and the hot deformed at 46 HB. The hardness of the hot deformed CuMg alloys was also lower when compared to the cold deformed samples. The lower hardness of hot deformed non-ferrous metals was explained by many authors as the increase of temperature, which leads to the migration of grain boundaries as well as the movement of dislocations and consequently, promotes the softening effects [58,59,60]. The lack of strain hardening of the hot deformed samples and the presence of solid-solution hardening is visible when assessing the hardness after hot upsetting, as the results are almost identical as in the non-deformed as-cast state samples [31]. The hardness of samples after cold upsetting tests differs in the case of CuMg alloys. The hardness of recrystallized and supersaturated samples is significantly lower than cold drawn samples after applying cold deformation of 50%. The difference is approximately 20 HB regardless of Mg content. This means that by applying cold drawing with a unit strain deformation (λ) of 1.2, the hardness of cold deformed samples increases quasi-linearly in the function of Mg content by 10–15%. Higher hardness of materials after prior cold drawing is linked to plastic deformation during which the increasing dislocations within the grains undergo interactions and lead to the formation of dislocation pile-up, which will impede the motion of dislocations. From the macroscopic point of view, the resistance to deformation increases and thus the strength of the materials increases as well [51,61].

The obtained results suggest that CuMg alloys are of high strength and reasonable electrical conductivity. Additionally, by adding Mg as an alloying additive, the density of the alloy will be lower than currently used alloys for high strength applications such as CuAg, CuNiSi, or CuZr alloys. The electrical conductivity of CuMg alloys is lower than CuAg alloys; however, the high price of silver limits the common use of these materials. Nowadays, high-strength copper alloys are mainly used as trolley wires and contact lines, cable terminals and connectors, brackets and handles for railway catenary systems, and applications that require high abrasive resistance such as 3D printing nozzles, welding caps, and tips [31]. The applications in these fields of the industry are where the CuMg alloys might find the most use.

## 4. Conclusions

The aim of this paper was to verify the deformability of CuMg alloys in various states (supersaturated, cold drawn, recrystallized) in cold upsetting tests and additionally in hot upsetting for the as-cast state. The following conclusions were made:

The chemical composition of tested samples exhibited slightly lower than nominal Mg content. During the metallurgical synthesis, the amount of Mg was 5% higher than nominal, meaning that this amount of Mg must have evaporated during the process.

Applying the solutioning at 700 °C for 10 h caused the Mg-rich phases to dissolve in the case of CuMg alloys with up to 3 wt.% of Mg. Samples with higher Mg content exhibited fragmentation of Mg-rich phases after subjecting them to cold upsetting when the samples were in the cold drawn state. This might suggest limited deformability of two-phase CuMg alloys even after prior solutioning was applied.

After subjecting the CuMg alloys to solutioning, it was possible to conduct cold upsetting with 50% strain without cracks or irregularities at stress–strain curves, and with visible effects of work hardening. Therefore, the CuMg alloys can be subjected to cold deformation after solutioning or hot deformation in the as-cast state.

There was almost a twofold difference between the hot deformed samples and the cold deformed samples that were subjected to prior cold drawing in terms of material hardness. It was caused by the softening effect of high temperature during hot upsetting and increasing resistance to deformation during cold upsetting.

Since the tested materials can be subjected to further artificial ageing, the research can be further extended by applying various temperatures and times of ageing in order to assess the response of CuMg alloys to deformation after subjecting them to the precipitation-hardening process.

## Figures and Tables

**Table 1:** The nominal chemical composition of cast rods (wt.%) [28].

Element	Cu	Mg	Other
Cu	Minimum 99.9	Maximum 0.02	Max. 0.1
CuMg2	98 ± 0.05	2 ± 0.05	Max. 0.1
CuMg2.4	97.6 ± 0.05	2.4 ± 0.05	Max. 0.1
CuMg2.8	97.2 ± 0.05	2.8 ± 0.05	Max. 0.1
CuMg3	97 ± 0.05	3 ± 0.05	Max. 0.1
CuMg3.2	96.8 ± 0.05	3.2 ± 0.05	Max. 0.1
CuMg3.6	96.4 ± 0.05	3.6 ± 0.05	Max. 0.1
CuMg4	96 ± 0.05	4 ± 0.05	Max. 0.1

**Table 2:** Average values of measured chemical composition of the tested materials.

Element	Cu	Mg	Other
Cu	99.92 ± 0.011	0.001 ± 0.001	Bal.
CuMg2	98.0 ± 0.043	1.99 ± 0.025	Bal.
CuMg2.4	97.6 ± 0.031	2.37 ± 0.014	Bal.
CuMg2.8	97.2 ± 0.055	2.78 ± 0.027	Bal.
CuMg3	97.0 ± 0.049	2.99 ± 0.028	Bal.
CuMg3.2	96.8 ± 0.031	3.20 ± 0.021	Bal.
CuMg3.6	96.4 ± 0.038	3.58 ± 0.029	Bal.
CuMg4	96.0 ± 0.045	3.98 ± 0.026	Bal.

**Table 3:** The collective data of stress recorded at 50% of applied strain.

	Supersaturated	Supersaturated and Cold Drawn	Supersaturated, Cold Drawn, and Recrystallized	Hot Deformed at 700 °C
Group of materials	Engineering stress recorded at 50% of strain (MPa)			
Cu	508	559	608	64
Single-phase CuMg	949–970	961–992	903–941	116
CuMg3	1023	1049	1045	126
Two-phase CuMg	1136–1157	1130–1162	1137–1170	120

## Data Availability

The original contributions presented in the study are included in the article material; further inquiries can be directed to the corresponding author.

## References

[1] Saini D.K., Bafna N., Jha P.K. (2024). Effect of sheet thickness on the solidification and quality of Al-Mg2Si composite sheet fabricated by continuous casting. J. Manuf. Process..

[2] Bagherian E.-R., Fan Y., Cooper M., Frame B., Abdolvand A. (2016). Effect of melt temperature, cleanout cycle, continuous casting direction (horizontal / vertical) and super-cooler size on tensile strength, elongation percentage and microstructure of continuous cast copper alloys. Metall. Res. Technol..

[3] Strzępek P., Mamala A., Boumerzoug Z., Baudin T., Brisset F., Zasadzińska M., Noga P. (2024). Effect of Horizontal Continuous Casting Parameters on Cyclic Macrosegregation, Microstructure, and Properties of High-Strength Cu–Mg Alloy Cast Rod. Metall. Mater. Trans. A.

[4] Jiang M.-Y., Meng X.-Y., Zhou J., Wei Z.-Y., Zhang H.-F., Shen P. (2024). Design and manufacture of near-net-shape metal-ceramic composites with gradient-layered structure and optimized damage tolerance. Addit. Manuf..

[5] Fahlman B.D. (2023). Metals. Materials Chemistry.

[6] Avdeeva L.K., Godulyan L.V., Kovalev A.I., Wainstein D.L., Vakhrushev V.O. (2024). Corrosion properties, chemical composition, and surface morphology of non-ferrous metals after tests at different temperature and humidity conditions. Metallurgist.

[7] Zhu X., Chen Y., Chen J. (2021). Effects of non-ferrous metal prices and uncertainty on industry stock market under different market conditions. Resour. Policy.

[8] Smyrak B. (2023). Analysis of the quality of aluminum overhead conductors after 30 years of operation. Eng. Fail. Anal..

[9] Meyberg R.A., Absi Salas F.M., Domingues L.A.M.C., Correia de Barros M.T., Lima A.C.S. (2020). Experimental study on the transformer effect in an ACSR cable. Int. J. Electr. Power Energy Syst..

[10] Ma X., Gao L., Zhang J., Zhang L.-C. (2017). Fretting Wear Behaviors of Aluminum Cable Steel Reinforced (ACSR) Conductors in High-Voltage Transmission Line. Metals.

[11] Bochniak W., Korbel A., Ostachowski P., Lagoda M. (2018). Plastic flow of metals under cyclic change of deformation path conditions. Arch. Civ. Mech. Eng..

[12] Muszka K., Madej L., Majta J. (2013). The effects of deformation and microstructure inhomogeneities in the Accumulative Angular Drawing (AAD). Mater. Sci. Eng. A.

[13] Gronostajski Z., Hawryluk M. (2008). The main aspects of precision forging. Arch. Civ. Mech. Eng..

[14] Millenin A., Wróbel M., Kustra P. (2022). Investigation of the workability and surface roughness of thin brass wires in various dieless drawing technologies. Arch. Civ. Mech. Eng..

[15] Liu S., Xie T., Han J., Shan X. (2022). Stress superposition effect in ultrasonic drawing of titanium wires: An experimental study. Ultrasonics.

[16] Leśniak D., Zasadziński J., Libura W., Gronostajcki Z., Śliwa R., Leszczyńska-Madej B., Kaszuba M., Woźnicki A., Płonka B., Widomski P. (2024). Latest advances in extrusion processes of light metals. Arch. Civ. Mech. Eng..

[17] Maleta M., Głuchowski W., Rdzawski Z., Łagoda M., Domagała-Dubiel J. (2023). Influence of the speed of downward semi-continuous casting on the crystal size and mechanical properties of recycled copper. Metalugija.

[18] Davis J.R. (2001). ASM Specialty Handbook: Copper and Copper Alloys.

[19] Nordheim L. (1931). Zur Elektronentheorie der Metalle. I. Ann. Phys..

[20] Shariyari F., Shaeri M.H., Dashti A., Zarei Z., Noghani M.T., Cho J.H., Djavanroodi F. (2022). Evolution of mechanical properties, microstructure and texture and of various brass alloys processed by multi-directional forging. Mater. Sci. Eng. A.

[21] Brudny A., Kulasa J., Cwolek B., Malec W., Juszczyk B. (2022). Influence of the continuous casting process of tin-zinc-lead bronze on the wear of the graphite crystallizer. Metalurgija.

[22] Ma S., Ye C., Yang X., Fu L., Fan J., Wang J., Shan A. (2023). Microstructure evolution during the heavy warm rolling of a nickel aluminum bronze. Mater. Sci. Eng. A.

[23] Zhang R., Li Z., Sheng X., Gao Y., Lei Q. (2020). Grain refinement and mechanical properties improvements in a high strength Cu–Ni–Si alloy during multidirectional forging. Fusion Eng. Des..

[24] Tardieu S., Mesguich D., Lonjon A., Lecouturier F., Ferreira N., Chevallier G., Proietti A., Estournes C., Caurent C. (2019). Nanostructured 1% silver-copper composite wires with a high tensile strength and a high electrical conductivity. Mater. Sci. Eng. A.

[25] Dölling J., Henle R., Prahl U., Zilly A., Nandi G. (2022). Copper-Based Alloys with Optimized Hardness and High Conductivity: Research on Precipitation Hardening of Low-Alloyed Binary CuSc Alloys. Metals.

[26] Ma B., An B., Zhao X., Li Y., Du J., Wang E. (2023). Microstructure and properties of Cu-Cr-Zr alloy by doping Sc. Mater. Lett..

[27] Yu X., Hou Y., Ren X., Sun C., Wang M. (2022). Research progress on the removal, recovery and direct high-value materialization of valuable metal elements in electroplating/electroless plating waste solution. J. Water Proc. Eng..

[28] Copper Alloys Knowledge Base. http://www.conductivity-app.org/.

[29] Rodriguez-Calvillo P., Ferrer N., Cabrera-Marrero J.M. (2015). Analysis of microstructure and strengthening in CuMg alloys deformed by equal channel angular pressing. J. Alloys Compd..

[30] Gorsse S., Ouvard B., Goune M., Poulon-Quintin A. (2015). Microstructural design of new high conductivity–high strength Cu-based alloy. J. Alloys Compd..

[31] Strzępek P., Zasadzińska M. (2024). Prospective cold metal working and analysis of deformation susceptibility of CuMg alloys with high magnesium content. Sci. Rep..

[32] Fan J., Liu Z., Liu W., Wang C. (2023). Simulation and Experiment Study on Cone End Billet Method in Upsetting Billet with a Large Height-to-Diameter Ratio. Appl. Sci..

[33] Schubert K., Anderko K. (1951). Crystal Structure of the NiMg2, CuMg2, and AuMg3. Z. Met..

[34] Spriggs G.E., Beiss P., Ruthardt R., Warlimont H. (2005). Properties of diamond and cubic boron nitride. Powder Metallurgy Data. Refractory, Hard and Intermetallic Materials.

[35] Dhondapure P., Rajakrishnan N., Nayak S., Champliaud H., Morin J.-B., Jahazi M. (2024). Influence of deformation path on microstructure evolution during the open die forging of large size ingot of high strength steel: Experiments and FE analysis. Int. J. Adv. Manuf. Technol..

[36] Mohammadi H., Eivani A.R., Seyedein S.H., Ghosh M., Jafarian H.R. (2023). Evolution of dynamic recrystallization behavior and simulation of isothermal compression of Zn–22Al alloy. J. Mater. Res. Technol..

[37] Wang Z.J., Cheng L.D. (2009). Experimental research and numerical simulation of the dynamic cylinder upsetting. Mater. Sci. Eng. A.

[38] Hamzah S., Ståhlberg U. (1998). A study of pore closure in the manufacturing of heavy rings. J. Mater. Process. Technol..

[39] Lee M.C., Jang S.M., Cho J.H. (2008). Finite element simulation of pore closing during cylinder upsetting. Int. J. Mod. Phys. B.

[40] Xu Y., Chen C., Zhang X., Dai H., Jia J., Bai Z. (2018). Dynamic recrystallization kinetics and microstructure evolution of an AZ91D magnesium alloy during hot compression. Mater. Charact..

[41] Yang Q.-S., Duan J.-X., Deng A.-Y., Wang E.-G. (2024). Numerical simulation of macrosegregation phenomenon in Cu-6wt%Ag alloy ingots fabricated by electromagnetic stirring. J. Mater. Res. Technol..

[42] Lesoult G. (2005). Macrosegregation in steel strands and ingots: Characterisation, formation and consequences. Mater. Sci. Eng. A-Struct..

[43] Prescott P., Incropera F.P. (1996). Convection heat and mass transfer in alloy solidification. Adv. Heat Tranf..

[44] Trofimov D.M., Imayev V.M., Imayev R.M. (2023). Influence of upset forging and heat treatment on the microstructure and mechanical properties of a new β-solidifying γ-TiAl alloy. Intermetallics.

[45] Wang J., Langlois L., Rafiq M., Bigot R., Lu H. (2014). Study of the hot forging of weld cladded work pieces using upsetting tests. J. Mater. Process. Technol..

[46] Jedrasik P., Shercliff H. (2021). Finite element analysis of small-scale hot compression testing. J. Mater. Sci. Technol..

[47] Maki K., Ito Y., Matsunaga H., Mori H. (2013). Solid-solution copper alloys with high strength and high electrical conductivity. Scr. Mater..

[48] Zhou M., Tang S., Zhang Y., Zheng X., Xu D., Zhang Z., Li Y., Fu L., Li X., Tian B. (2023). Ce effects on dynamic recrystallization and microstructure of the copper alloy under hot deformation behavior with different strain rate. J. Mater. Res. Technol..

[49] Shen K., Guo M., Wang M. (2010). Recrystallization characteristics of a fine grained copper alloy. Mater. Chem. Phys..

[50] Hollomon J.H. (1945). Tensile Deformation. Trans. Tms-AIME.

[51] Wang X., Xiao Z., Zhou T., Jiang X. (2024). Deformation mechanism and properties evolution of a copper alloy with ultra-high strength after cold drawing and subsequent aging treatment. Mater. Sci. Eng. A.

[52] Lalpoor M., Eskin D.G., Ruvalcaba D., Fjær H.G., Ten Cate A., Ontijt N., Katherman L. (2011). Cold cracking in DC-cast high strength aluminum alloy ingots: An intrinsic problem intensified by casting process parameters. Mater. Sci. Eng. A.

[53] Jiang J., Tong Z., Huang M., Wang Y., Zhao W. (2024). Effect of recrystallization annealing on microstructure and properties of cold-deformed CoCrCu1.2FeNi high entropy alloy. J. Alloys Compd..

[54] Li X., Zhang Q., Wenpeng L., Liang J., Gu S. (2023). Microstructure and Texture of Pure Copper under Large Compression Deformation and Different Annealing Times. Coatings.

[55] Rezaei Ashtiani H.R., Shayanpoor A.A. (2022). Effect of Initial Grain Size on the Hot Deformation Behavior and Microstructural Evolution of Pure Copper. Acta Metall. Sin. Engl. Lett..

[56] Çetinarslan C.S. (2009). Effect of cold plastic deformation on electrical conductivity of various materials. Mater. Des..

[57] Freudenberger J., Kauffmann A., Klauß H., Marr T., Nenkov K., Subramanya Sarma V., Schultz L. (2010). Studies on recrystallization of single-phase copper alloys by resistance measurements. Acta Mater..

[58] Chen T., Wen M., Cui H., Guo J., Wang C. (2023). Hot Deformation Behavior and Processing Maps of Pure Copper during Isothermal Compression. Materials.

[59] Prasad Y.V.R.K., Rao K.P. (2004). Mechanisms of high temperature deformation in electrolytic copper in extended ranges of temperature and strain rate. Mater. Sci. Eng. A.

[60] Yang J.Y., Kim W.J. (2020). Examination of high-temperature mechanisms and behavior under compression and processing maps of pure copper. J. Mater. Res. Technol..

[61] Mao Q., Liu Y., Zhao Y. (2024). A review on copper alloys with high strength and high electrical conductivity. J. Alloys Compd..

